# The effect of HMGA1 in LPS-induced Myocardial Inflammation

**DOI:** 10.7150/ijbs.39947

**Published:** 2020-03-26

**Authors:** Zhu-Lan Cai, Bo Shen, Yuan Yuan, Chen Liu, Qing-Wen Xie, Tong-Tong Hu, Qi Yao, Qing-Qing Wu, Qi-Zhu Tang

**Affiliations:** 1 Department of Cardiology, Renmin Hospital of Wuhan University, Wuhan 430060, RP China.; 2 Hubei Key Laboratory of Metabolic and Chronic Diseases, Wuhan 430060, RP China.

**Keywords:** Myocardial inflammation, lipopolysaccharide, HMGA1, cyclooxygenase-2, STAT3

## Abstract

**Aims**: The High Mobility Group A1 (HMGA1) proteins, serving as a dynamic regulator of gene transcription and chromatin remodeling, play an influential part in the pathological process of a large number of cardiovascular diseases. However, the precise role of HMGA1 in sepsis induced cardiomyopathy (SIC) remains unintelligible. This research was designed to illustrate the effect of HMGA1 involved in SIC.

**Methods and Results**: Cardiomyocyte-specific HMGA1 overexpression was obtained using an adeno-associated virus system with intramyocardial injection in mice heart. The model of SIC in mice was constructed via intraperitoneal injection of lipopolysaccharide (LPS) for 6h. H9c2 rat cardiomyocytes was stimulated with LPS for 12h. HMGA1 expression was upregulated in murine inflammatory hearts as well as LPS stimulated H9c2 cardiomyocytes. HMGA1-overexpressing exhibited aggravated cardiac dysfunction, cardiac inflammation as well as cells apoptosis following LPS treatment both in vivo and *in vitro* experiment. Interestingly, HMGA1 knockdown in H9c2 cardiomyocytes attenuated LPS-induced cardiomyocyte inflammation, but aggravated cell apoptosis. Mechanistically, we found that overexpression of HMGA1 induced increased expression of cyclooxygenase-2 (COX-2). COX-2 inhibitor alleviated the aggravation of inflammation and apoptosis in HMGA1 overexpressed H9c2 cardiomyocytes whereas HMGA1 knockdown induced a reduction in signal transducer and activators of transcription 3 (STAT3) expression. STAT3 agonist reversed HMGA1 silence induced anti-inflammatory effects, while ameliorated cell apoptosis induced by LPS.

**Conclusion**: In conclusion, our results suggest that overexpression of HMGA1 aggravated cardiomyocytes inflammation and apoptosis by up-regulating COX-2 expression, while silence of HMGA1 expression attenuated inflammation but aggregated cell apoptosis via down-regulation of STAT3.

## Introduction

Sepsis refers to complex systemic inflammatory response syndrome (SIRS) caused by infection. Due to the high incidence, mortality and hospitalization costs by sepsis, attention to SIRS is increasingly paid due. Severe sepsis refers to a life-threatening organ dysfunction with poor tissue perfusion, or hypotension 1. Among them, sepsis-induced cardiomyopathy (SIC) produces deadly injury to patients with sepsis. SIC with severe cardiac dysfunction, manifested by reduced blood pressure; decreased left ventricular ejection fraction; reduced short-axis shortening; refractory heart failure and systolic diastolic dysfunction; is a main reason of complication and high mortality in ICU patients 2.The pathogenesis of SIC is multifactorial and involves mitochondrial dysfunction, disorder of calcium regulation, oxidative stress, dysregulation of inflammatory cytokines, autonomic nervous system dysregulation, apoptosis and injury of endothelial cells 3. In recent years, with the development of cardiac function monitoring technology, especially the wide application of bedside echocardiography, people have a new understanding of SIC. However, the specific mechanism of SIC has not yet been fully elucidated.

High mobility group (HMG) proteins refer to a superfamily of nonhistone proteins involved in cell chromatin remodeling 4, which were named for their rapid electrophoretic mobility in polyacrylamide gels 5. It is reported that HMGA1, as a member of HMG, was found in the human cervical cancer cell line Hela with high malignant proliferation ability 6,7. In addition, HMGA1 is highly expressed during a variety of biological embryogenesis and is widely overexpressed in diverse tumors, but has little to no expression in most adult and differentiated tissues 8. HMGA1 proteins bind the minor groove of AT-rich DNA sequences through its unique functional motifs, called ''AT-hooks'', located at the NH_2_-terminal region of HMGA1 to transform chromatin structure and recruit additional transcription factors 9. By interacting with both DNA and transcription factors, HMGA1 participates in the regulation of a multitude of fundamental cellular biological processes including cell cycle regulation 10, embryonic development 11, tumor transformation 4, cell proliferation and differentiation 12, apoptosis 13, energy metabolism 14 and repair of DNA 15.

In addition, it has been proven that HMGA1 also contributes to a multitude of cardiovascular diseases, including coronary microembolization 16, myocardial infarction 17 and cardiomyocyte hypertrophy 18. Nevertheless, studies concerning the function of HMGA1 in SIC are still lacking. In the current study, we constructed the model of LPS-induced sepsis in mice heart and H9c2 cardiomyocytes to explore the effects of HMGA1 in SIC.

## Materials and Methods

### Materials

Adeno-associated virus 9 (AAV9) carrying HMGA1 (AAV9-HMGA1) (NM_145901) or green fluorescent protein (AAV9-GFP) was constructed by Vigene Bioscience (Shangdong, China). HMGA1 siRNA (si-HMGA1) and siRNA negative control (si-NC) were purchased from Ribobio Co., Ltd (Guangzhou, China). Lipo6000 ™ transfection reagent was purchased from Beyotime Biotechnology (Shanghai, China). LPS from Escherichia coli O55:B5 was bought from Sigma. The primary antibodies that interact with the proteins listed below were purchased from Cell Signaling Technology: Cytochrome C, Bcl-2-associated X protein (Bax), B-cell lymphoma 2 (Bcl-2), P-STAT3, STAT3, COX-2 and glyceraldehyde-3-phosphate dehydrogenase (GAPDH). The followed primary antibodies were obtained from ABCAM: HMGA1. All the secondary antibodies appearing in the research were purchased from LI-COR Biosciences. The BCA protein assay kit was from Pierce Biotechnology Inc at Rockford. ApopTag® Plus *In situ* Apoptosis Fluorescein Detection Kit (S7111) was bought from Millipore Corporation.

### Animals and models

Operational process in the experiment were conducted in compliance with the Guidelines for the Care and Use of Laboratory Animals published by the United States National Institutes of Health (NIH Publication, revised 2011) and were proved by the Chinese Animal Welfare Committee as well as Animal Care and Use Committee of Renmin Hospital of Wuhan University (approval number: WDRX-2018K010).

C57BL/6 adult male mice weighing 23.5-27.5 g and aged 8-10 weeks were purchased from Institute of Laboratory Animal Science, Chinese Academy of Medical Sciences (Beijing, China). One week before the start of the experiment, all mice were housed in the SPF-level animal room of the Institute of Cardiovascular Diseases, Renmin Hospital of Wuhan University (Wuhan, China), with free access to water and food. The temperature of the living environment for the mice was set to 20-25°C, the humidity was controlled at 50±5%, and the light illumination was alternated for 12h. The mice were divided into the AAV9-GFP+Saline group; AAV9-HMGA1+ Saline group; AAV9-GFP +LPS group; and the AAV9-HMGA1+LPS group (n=15), in the light of a random number table. Mice in AAV9-HMGA1 group received a single intramyocardial injection with AAV9-HMGA1 for the dose of 1×10^11^ viral genome particles/mouse, while the mice in AAV9-GFP group were subjected to AAV9-GFP. In order to build a model of acute SIC, mice were received with LPS dissolved in sterile saline for the dose of 6 mg/kg via single intraperitoneal injection after transfection of AAV9-HMGA1 or AAV9-GFP for one week. For the saline group, we gave an equal volume of sterile saline. The dosage of LPS was selected on the basis of reports in the literature, which could bring about significant myocardial inflammation 19. Cardiac function was evaluated by echocardiography 6h after the injection of LPS or sterile saline. The mice were then sacrificed via cervical vertebra dislocation. After rapid thoracotomy, the intact heart was rapidly excised to be fixed in 10% formaldehyde solution and embedded in paraffin or left ventricular tissue is collected, rapidly frozen in liquid nitrogen, and stored in a -80°C refrigerator for further use.

### Echocardiography

Transthoracic echocardiography was performed 6h after injecting with LPS intraperitoneally. The mice were anesthetized by inhalation of isoflurane and were subjected to echocardiography using a Mylab 30CV ultrasound diagnostic apparatus (Esaote S.P, A., Genoa, Italy) equipped with a 15 MHz linear array ultrasound transducer. The short-axis standard view of the left ventricular papillary muscle was selected and measured. Heart rate (HR), left ventricular end-systolic diameter (LVESD) and left ventricular end-diastolic diameter (LVEDD) were recorded. Left ventricular ejection fraction (LVEF) and left ventricular fractional shortening (LVFS) were also calculated.

### Western blotting and quantitative real-time PCR

Samples of each group were dissolved in lysis buffer, and the supernatant was taken by high speed centrifugation (12000 g, 4°C) following ultrasonic lysis. The protein concentration was measured by the BCA Protein Assay Kit (Waltham, MA, USA) and unified. Proteins from heart tissue or cultured cardiomyocytes were subjected to sodium dodecyl sulfate polyacrylamide gel electrophoresis, and the separated proteins were transferred to polyvinylidene fluoride membranes (Millipore, Billerica, MA, USA). It was blocked with 5% skim milk for 1h in the condition of 25°C, then allowed to interact with the indicated primary antibody overnight at 4°C, and incubated with the secondary antibody for 1h at 37°C on the next day. PVDF membranes with target protein were detected and analyzed by an Odyssey infrared two-color fluorescence system (LI-COR Biosciences, USA). All of the protein expression levels were quantified using Image J software and normalized to GAPDH that was an internal reference protein.

The mRNA expression of HMGA1, tumor necrosis factor-α (TNF-α), interleukin-1 (IL-1), interleukin-6 (IL-6) in heart tissue or cardiomyocytes were detected by Real-Time PCR (RT-PCR) according to our previous work 20. Briefly, total RNA was extracted by TRIZOL (Invitrogen) and the quality of RNA extraction was evaluated by spectrophotometer A260/A280 and A230/A260 ratio. The mRNA was reverse transcribed into cDNA in line with the instructions of First Strand cDNA Synthesis Kit (Roche, Switzerland) and LightCycler@ 480 SYBR Green l Master Mix (Roche) was used to amplify the cDNA. The transcriptional level of each indicator was normalized with GAPDH as an internal reference gene, and the relative expression levels of each group compared with the control group were calculated. Primer sequences employed in this research are listed in Table 1.

### Cell culture and treatments

We obtained the rat cardiomyocyte-derived cell line H9c2 from the Cell Bank of the Chinese Academy of Sciences (China). H9c2 was cultured in DMEM medium containing 10% FBS in the incubator (SANYO 18M, Japan) of 37℃ with 5% CO_2_. When the cell density reached 70% to 80%, the cells were passaged with 1mL of 0.25% Trypsin-EDTA (GIBCO) and seeded in six-well plates for Western blots and RT-RNA analysis, in twenty-four-well plates for TUNEL staining and Immunofluorescence staining, and in ninety-six-well plates for CCK8 measurement. After 24-48h, the current medium was substituted with DMEM basic medium without fetal bovine serum to starve cells for 12h. Then, the cells were transfected with AAV9-HMGA1 for 6-8h to overexpress HMGA1, and the control group was transfected with AAV9-GFP. Transfection of siRNA was implemented using Lipo6000™ transfection reagent on the basis of the manufacturer's instructions. Subsequently, the cells were stimulated with LPS at a final concentration of 1µg/ml for 12h to induce cardiac inflammatory response *in vitro*.

### Cell Counting Kit-8 (CCK-8)

We used the CCK8 assay kit to evaluate the viability of treated cell. 100μL cell suspension was dispensed in a 96-well plate and the cell received different treatments according to experimental design. Then, the treated cells were added with 10μL of CCK-8 solution per well and continued to be cultured in an incubator for 4h. Cell viability was measured with a microplate reader for the absorbance at a wavelength of 450 nm. The influence of HMGA1 on cell viability with LPS stimuli was calculated through the following formula:

Cell viability (%) = OD_treatment_/OD_control_

### TdT-mediated dUTP nick end-labelling (TUNEL) staining

TUNEL staining was administrated to observe the apoptosis index both in vivo and *in vitro* based on the standard protocol using ApopTag® Plus Fluorescein *in situ* Apoptosis Detection Kit (Millipore, Billerica, MA, USA).The cells were executed with 1% paraformaldehyde for 10min in the condition of 25°C and were treated with a mixture of ethanol and acetic acid for 5min. Dewaxed hydrated heart tissue was incubated with proteinase K for 15min. Subsequently, the cells or tissue were supplemented with the equilibration buffer at room temperature, then, treated with TdT Enzyme working solution and Stop/WashBuffer working solution. After the cells or tissue were incubated with Anti-Digoxigenin Fluorescein working solution for 30min in the dark, SlowFade Gold Antifade Reagent (with DAPI) was used to seal.

### Immunohistochemistry staining

After dehydration in ethanol, the hearts tissues embedded in paraffin were placed in citrate for antigen repairing. Subsequently the heart tissue was were incubated with 3% H_2_O_2_ for 10min under the condition of 25°C and treated with 10% goat serum for 30min at 37°C, following by incubation with indicated antibodies overnight at the temperature of 4°C. On the next day, the tissue was treated with the corresponding secondary antibody for 30min. The DAB working solution was added under an optical microscope to control the display time. Afterwards, the nucleus is stained with hematoxylin. Alcohol and xylene are used to dehydrate and degrease the tissue.

### Immunofluorescence staining

Cell coverslips were fixed with 4% formaldehyde at room temperature for 15min, and then permeabilized in 0.2% TritonX-100 for 10min. Subsequently, cell coverslips were incubated with the primary antibody (1: 100; diluted in PBS) at 4°C overnight after being treated with 8% bovine serum at 37°C for 1h. The next day, the Alexa Fluor 488-goat anti-rabbit secondary antibody (1: 200; diluted with PBS) was used to probe target proteins. Finally, 4,6-diamidino-2-phenylindole (DAPI) was used for nuclear staining. The fluorescence microscope (Olympus, Tokyo, Japan) was employed to capture image.

### Statistical analysis

All quantitative data in this experiment were expressed as mean ± standard deviation (SD) and statistically analyzed by SPSS22.0 software. Comparisons between multiple groups were assessed via one-way variance analysis followed by Tukey post hoc test. While, comparisons between two groups were evaluated using Student's unpaired t-test. The difference between the groups was considered to be statistically significant under the condition that the P value was less than 0.05.

## Results

### HMGA1 expression of heart tissue or cardiomyocytes increased following LPS stimuli

**Figure 1:**
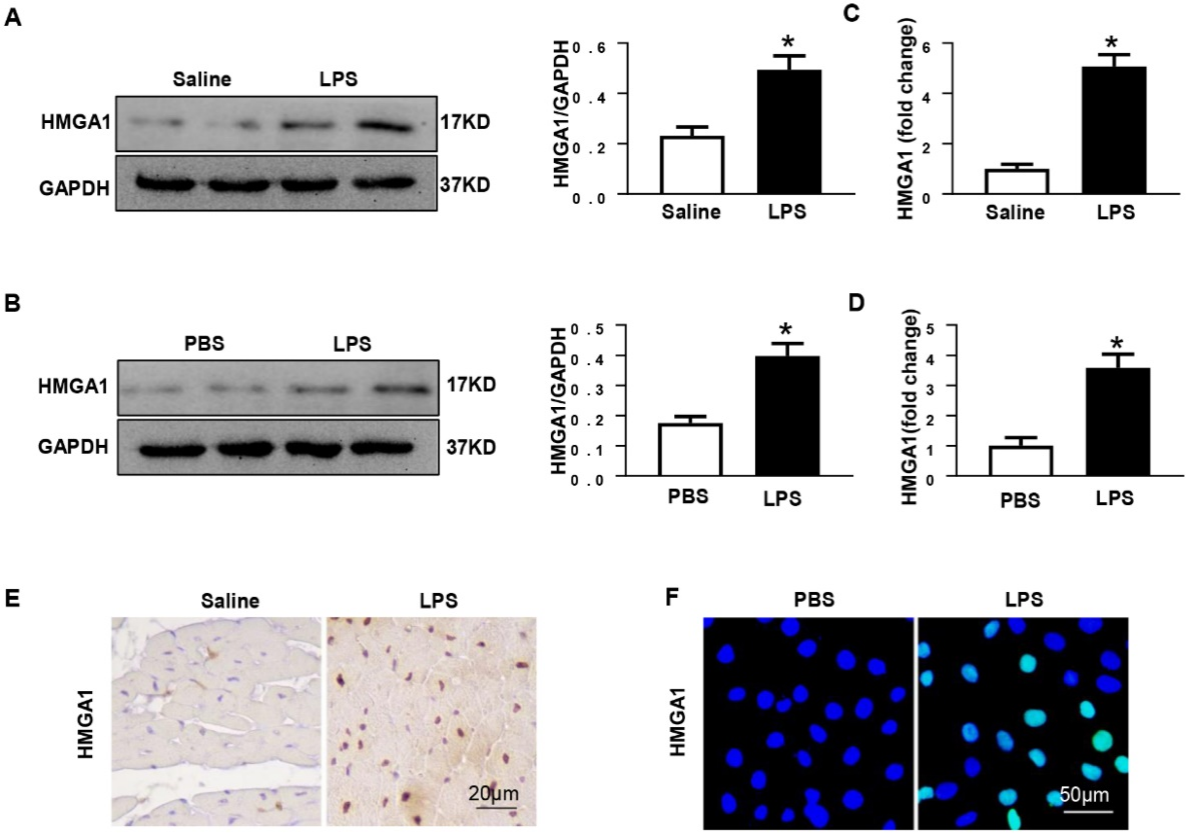
** HMGA1 expression of heart tissue or cardiomyocytes up-regulated after LPS stimuli. (A)** Protein expression level of HMGA1 in mice heart with or without LPS stimuli was shown in western blotting and was normalized to GAPDH (n=6). **(B)** Representative images of western blotting of HMGA1 expression in H9c2 cardiomyocytes and the protein expression level was normalized to GAPDH (n=6). **(C)** Cardiac HMGA1 mRNA expression was detected by PCR (n=6). **(D)** HMGA1 mRNA expression of H9c2 cardiomyocytes was examined by PCR (n=6). **(E)** HMGA1 expression in heart tissue was detected by immunohistochemistry staining (n=6). **(F)** HMGA1 expression in H9c2 cardiomyocytes was revealed by immunofluorescence staining (n=6). *P<0.05, vs. control group. Data were analyzed using Student's *t*-test.

Previous researches have shown that HMGA1 participated in the process of inflammatory responses. In order to detect the protein levels of HMGA1, mice heart tissues were collected after 6h of intraperitoneal injection with LPS at 6mg/kg. Western blotting illustrated that the expression level of HMGA1 significantly up-regulated in the mice hearts after the LPS stimuli compared with that in the hearts of control mice (Figure 1A). The mRNA level of HMGA1 increased in cardiac tissues with the induction of inflammation (Figure 1C). Immunohistochemical staining results of heart tissue sections demonstrated elevated levels of HMGA1 expression in inflammatory heart tissues (Figure 1E). The expression of HMGA1 in H9c2 cardiomyocytes challenged with LPS was estimated by Western Blot, RT-PCR and Immunofluorescence staining. Consistent with the results in vivo, there was a remarkable growth of HMGA1 expression in H9c2 cardiomyocytes after LPS stimulation in comparison with control group as shown in Figure 1.

### HMGA1 promoted cardiomyocytes inflammation and apoptosis induced by LPS

**Figure 2:**
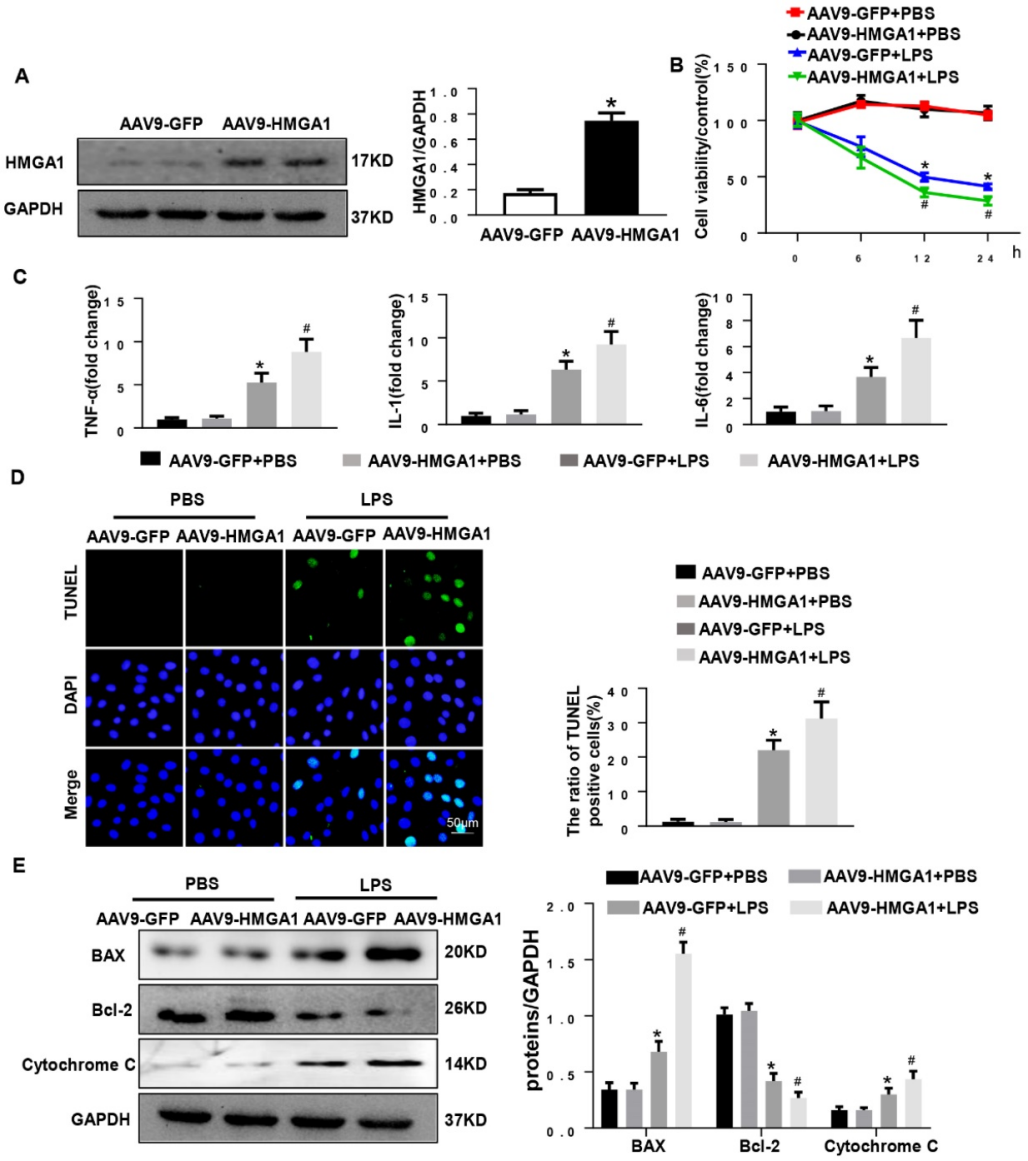
** HMGA1 promoted cardiomyocytes inflammation and apoptosis induced by LPS. (A)** The protein expression level of HMGA1 in the H9c2 cardiomyocytes following adeno-associated virus infection was normalized to GAPDH (n=6). **(B)** The viability of each group was detected by CCK8 (n=6). **(C)** RT-PCR for mRNA expression of inflammatory cytokines in H9c2 cardiomyocytes, such as TNF-α, IL-1, IL-6 (n=6). **(D)** TUNEL staining exhibited the cellular apoptosis in H9c2 cardiomyocytes after infection of AAV9-GFP or AAV9-HMGA1 vector with or without LPS stimuli (n=6). **(E)** Western blotting analyses of the apoptosis-related proteins, including Bax, Bcl-2 and Cytochrome C. and the protein expression level was normalized to GAPDH (n=6). *P<0.05, vs. AAV9-GFP+PBS group. ^#^*P*<0.05, vs. AAV9-GFP+ LPS group. The data are expressed as the mean±standard deviation (SD) and were compared by one-way ANOVA with Tukey post hoc analysis.

To further explore the effects of HMGA1 in myocardial inflammation, AAV9-HMGA1 was administrated to overexpress HMGA1 in H9c2 cardiomyocytes. Later, the expression of HMGA1 in cardiomyocytes was detected by western blotting. The result of the experiment showed that the level of HMGA1 significantly grew in H9c2 after transfection with AAV9-HMGA1 compared with the control group (Figure 2A). Then, we used LPS to induce cellular inflammatory responses. In comparison with the control group, the cell viability in H9c2 cells overexpressing HMGA1 was significantly lower with LPS stimuli (Figure 2B).

We also assessed the influence of HMGA1 on the production of cytokines (TNFα, IL-1β and IL-6) in LPS-treated H9c2 cells. Data indicated that mRNA expression levels of TNFα, IL-1 and IL-6 obviously up-regulated with dispose of LPS stimuli, which was evidently augmented by overexpressing of HMGA1 (Figure 2C). These data indicated that HMGA1 exacerbated LPS-induced inflammatory responses *in vitro*.

Additionally, TUNEL staining was employed to make an inquiry of the effect of HMGA1 on apoptosis in H9c2 cardiomyocytes with LPS stimulation. It was indicated that H9c2 cardiomyocytes treated with LPS triggered a significant increase in apoptosis and the overexpression of HMGA1 strikingly accelerated the apoptosis (Figure 5D). Furthermore, we further examined the protein levels of apoptotic markers including Bax, Bcl-2 and Cytochrome C in H9c2 cardiomyocytes. We found that H9c2 cardiomyocytes with exposure of LPS resulted in increasing the protein levels of pro-apoptotic Bax, Cytochrome C and reducing the protein level of anti-apoptotic Bcl-2. Furthermore, overexpression of HMGA1 in H9c2 cardiomyocytes could exacerbate these alterations (Figure 5E).

### Cardiac HMGA1-overexpression mice subjected to LPS exhibited cardiac dysfunction

**Figure 3:**
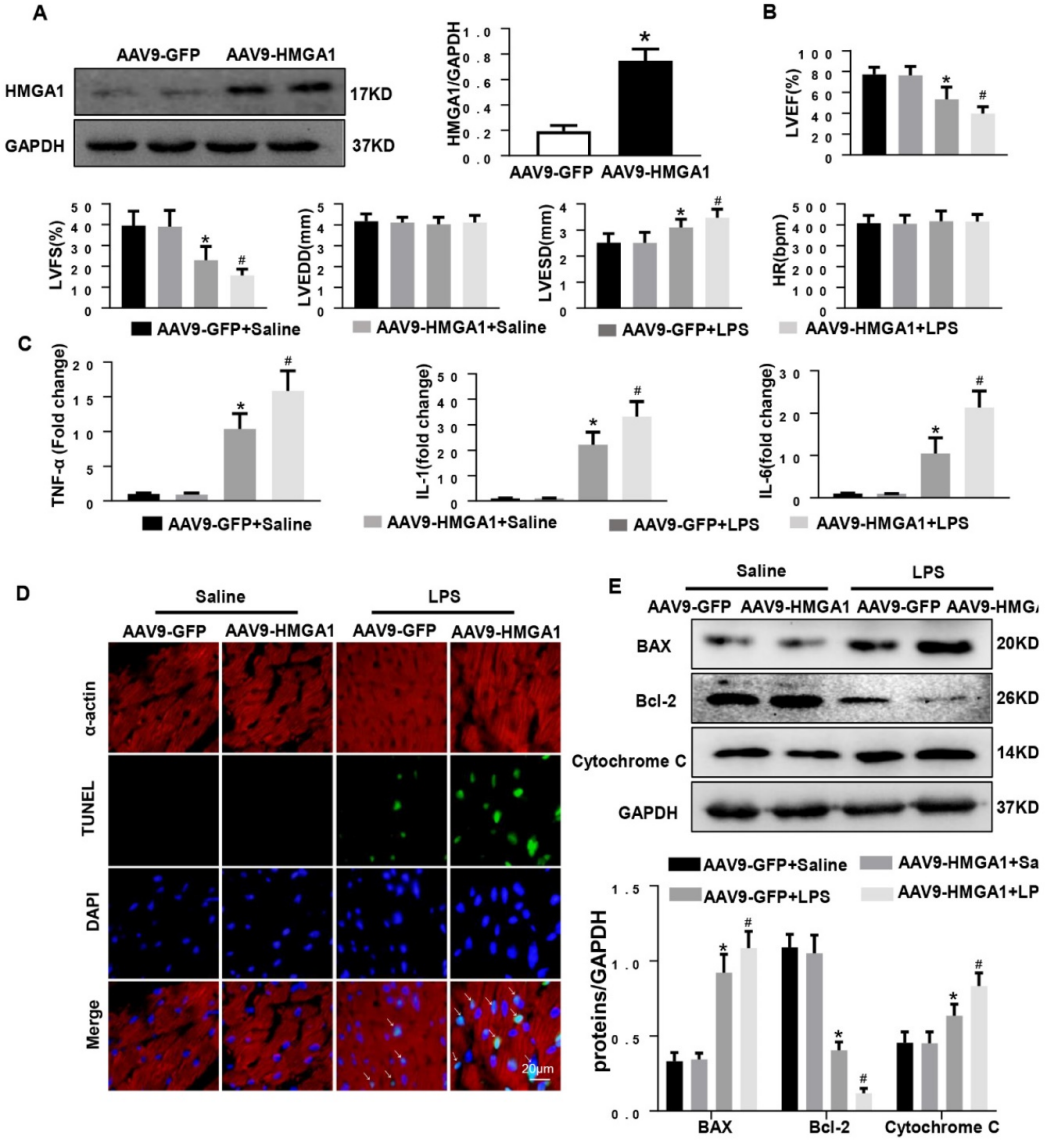
** Cardiac HMGA1-overexpression mice subjected to the treatment of LPS exhibited cardiac dysfunction. (A)** Immunoblots for HMGA1 and GAPDH following intramyocardial injection of AAV9-GFP or AAV9-HMGA1 vector(n=6) and HMGA1 expression was normalized to GAPDH.**(B)** Echocardiographic analysis of left ventricular ejection fraction (LVEF), left ventricular fractional shortening (LVFS), left ventricular end-systolic diameter (LVESD), left ventricular end-diastolic diameter (LVEDD) and heart rate(HR) 6h after injection of saline or LPS(n=10). **(C)** RT-PCR for mRNA level of inflammatory related genes in heart tissue, such as TNF-α, IL-1, IL-6 (n=6). **(D)** TUNEL staining showed the level of cell apoptosis in mouse heart tissue. **(E)** Representative western blotting images of the apoptosis-related proteins, including BAX, Bcl-2 and Cytochrome C. and the protein expression levels were normalized to GAPDH (n=6). *P<0.05, vs. CON group. ^#^*P*<0.05, vs. LPS group. The data are expressed as the mean±SD and were compared by one-way ANOVA with Tukey post hoc analysis.

To assess the effect of HMGA1 in SIC, we used intramyocardial injection of AAV9-HMGA1 to alter the expression of HMGA1 in cardiac tissue. One week later, the protein level of HMGA1 in mice heart was evaluated by western blotting. Our work suggested that the protein level of HMGA1 was significantly up-regulated in cardiac tissues injected with AAV9-HMGA1 compared to the control group (Figure 3A). Following the success of overexpression of HMGA1 in cardiac tissues after one week of intramyocardial injection, 6mg/kg LPS was given via intraperitoneal injection. After 6h, the echocardiographic were applied to investigate the cardiac function in SIC. As expected, the significant decrease in LVEF, LVFS, accompanying with the increase in LVESD, suggested more severe left ventricular dysfunction in HMGA1-overexpressing mice than the mice with LPS alone stimuli, but there was no difference in LVEDD and HR between the two groups (Figure 3B).

Subsequently, the mRNA expression levels of proinflammatory mediators including TNF-α, IL-1, IL-6 in cardiac tissues were detected by RT-PCR. The expression levels of proinflammatory cytokines were markedly increased after LPS injection. HMGA1 exacerbated the expression of proinflammatory cytokines (Figure 3C).

Inflammation induced by LPS resulted in cardiac dysfunction and cardiomyocyte apoptosis. To quest whether HMGA1 had influence on cardiomyocyte apoptosis after exposure with LPS, we performed TUNEL staining. As indicated in Figure 3D, there was an obvious increase in the TUNEL-positive myocardial cells in cardiac tissues challenged with LPS, and overexpression of HMGA1 in the heart evidently promoted the apoptosis with dispose of LPS. In addition, we examined the protein levels of Bax, Bcl-2 and Cytochrome C following stimulation of LPS with or without overexpression of HMGA1. Being consistent with the decrease in TUNEL-positive myocardial cells after stimulation with LPS, we found the expression of pro-apoptotic Cytochrome C, Bax upregulated and anti-apoptotic Bcl-2 downregulated after injection of LPS, and overexpression of HMGA1 could accelerated these alterations in response to LPS stimuli (Figure 3E).

### HMGA1 deficiency reduced cardiomyocyte inflammation, but aggravated apoptosis

**Figure 4:**
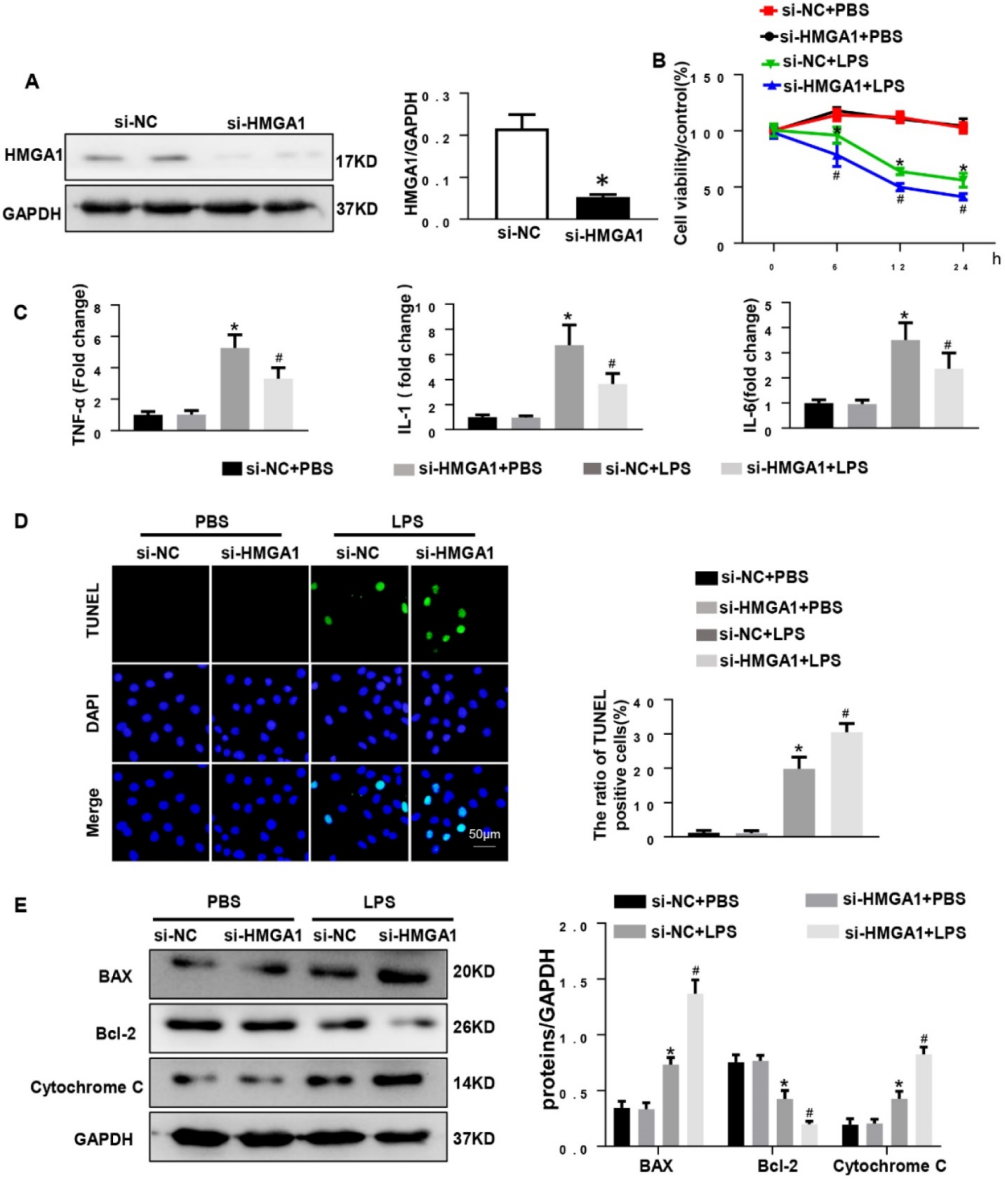
** HMGA1 deficiency reduced cardiomyocyte inflammation, but aggravated apoptosis. (A)** HMGA1 expression in the H9c2 cardiomyocytes after transfection with si-HMGA1 to knock down HMGA1 and protein expression level was normalized to GAPDH (n=6). **(B)** The viability of each group was detected by CCK8 (repeat 6 times). **(C)** RT-PCR for mRNA level of inflammatory cytokines in H9c2 cardiomyocytes, such as TNF-α, IL-1, IL-6 (n=6). **(D)** TUNEL staining exhibited the cellular apoptosis in H9c2 cardiomyocytes after transfection with siRNA or siHMGA1 with or without stimulation of LPS (n=6). **(E)** Western blotting analyses of the apoptosis-related proteins in H9c2 cardiomyocytes, such as Bax, Bcl-2 and Cytochrome C. and the protein expression level were normalized to GAPDH(n=6). *P<0.05, vs. si-NC+PBS group. ^#^*P*<0.05, vs. si-NC+LPS group. The data are expressed as the mean±SD and were compared by one-way ANOVA with Tukey post hoc analysis.

Given that overexpression of HMGA1 can aggravate LPS-induced inflammation, we hypothesized that inhibition of HMGA1 would attenuate inflammatory responses induced by LPS. We next used lipo6000 for transfection of small interfering RNA of HMGA1 (siHMGA1) to down-regulate the expression of HMGA1 in cardiomyocytes (Figure 4A). After that, H9c2 was incubated with LPS for 12h to induce cardiomyocytes inflammation. As we envisaged, PR-PCR analysis demonstrated transcriptional levels that were significantly decreased in response to LPS treatment in HMGA1 deficiency cardiomyocytes for TNF-α, IL-1 and IL-6 compared to control cardiomyocytes (Figure 4C). However, contrary to our assumptions, HMGA1 knockdown exacerbated the LPS-induced reduction of cell viability (Figure 4B). Moreover, the TUNEL-positive cells increased in the HMGA1-deficient group, indicating that HMGA1 deficiency enhanced LPS-induced cardiomyocyte apoptosis (Figure 4D). Additionally, consistent with TUNEL staining results, the expression of pro-apoptotic Cytochrome C, Bax significantly increased and anti-apoptotic Bcl-2 obviously decreased in inflammatory H9c2 cardiomyocytes subject to HMGA1 silence (Figure 4E).

### HMGA1 altered the expression of COX-2 and STAT3

**Figure 5:**
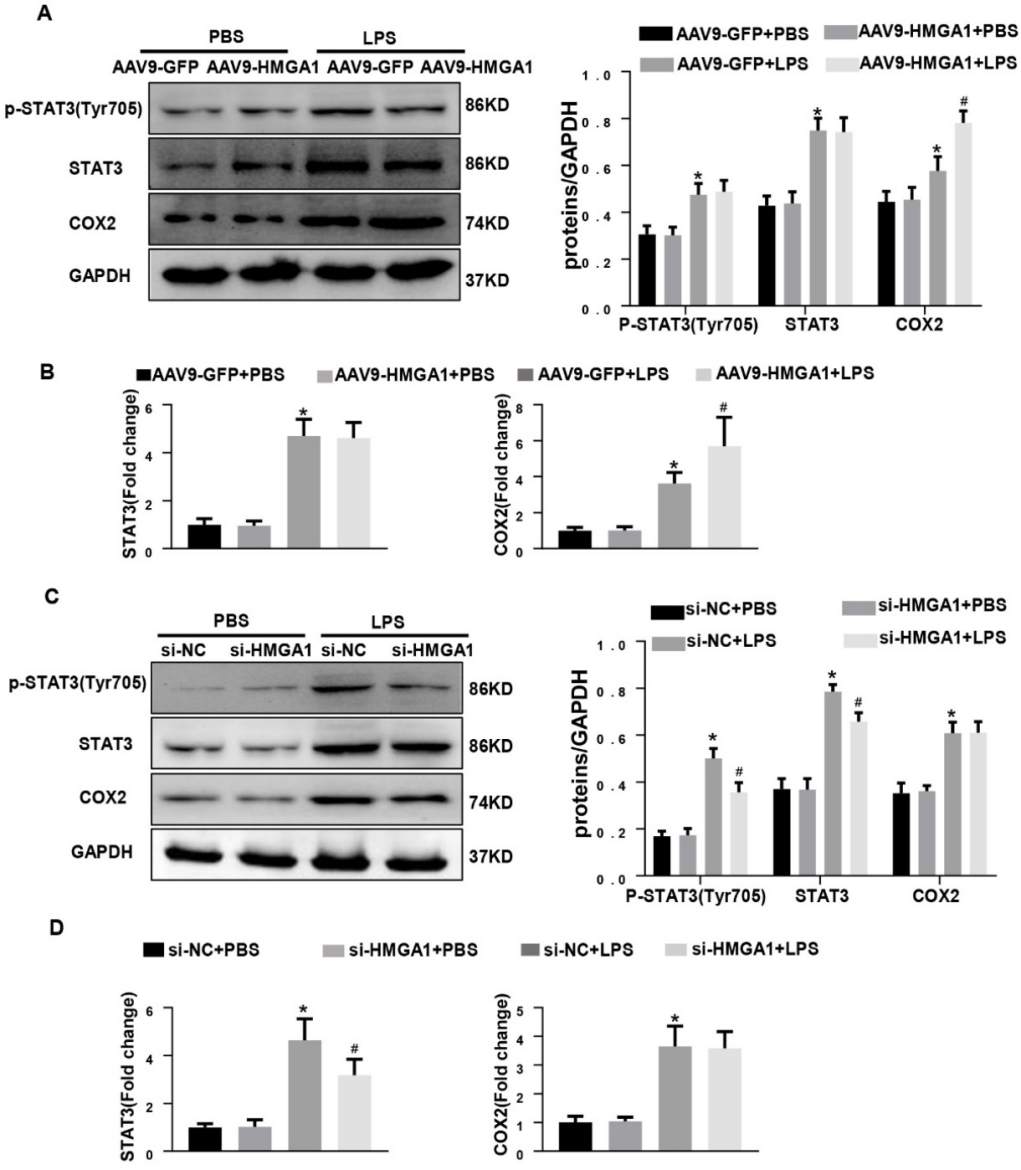
** HMGA1 altered the expression of COX-2 and STAT3. (A)** Representative western blotting images of P-STAT, STAT3 and COX-2 expression of H9c2 cardiomyocytes after infection of AAV9-GFP or AAV9-HMGA1 vector with or without LPS stimulation and the protein expression level was normalized to GAPDH(n=6). **(B)** RT-PCR for mRNA level of STAT3 and COX-2 after infection of AAV9-GFP or AAV9-HMGA1 vector with or without LPS stimuli and the expression level was normalized to GAPDH(n=6). **(C)** Representative western blotting images of P-STAT3, STAT3 and COX-2 expression of H9c2 cardiomyocytes after transfection of si-NC or si-HMGA1 with or without LPS treatment and all of the proteins were normalized to GAPDH(n=6). **(D)** RT-PCR for mRNA level of STAT3 and COX-2 after infection of si-HMGA1 or si-NC with or without LPS stimuli (n=6). *P<0.05, vs. AAV9-GFP/si-NC+PBS group. ^#^*P*<0.05, vs. AAV9-GFP/si-NC+LPS group. The data are represented as the mean±SD and compared by one-way ANOVA with Tukey post hoc analysis.

Previous studies have shown that HMGA1 activates COX-2 expression during tumorigenesis and STAT3 is a key downstream target of HMGA1.To examine whether HMGA1 regulated inflammatory response through altering the transcription of COX-2 or STAT3 after LPS stimuli, western blotting and PCR were used to detect the protein and transcription level of COX-2 and STAT3 in H9c2 cardiomyocytes with overexpression of HMGA1 or inhibiting the expression of HMGA1. Figure 5A demonstrated that the protein levels of COX-2 and STAT3 significantly increased in H9c2 following stimulation with LPS, and overexpression of HMGA1 in cardiomyocytes further enhanced the upregulation of COX-2 but did not affect the presentation of STAT3. PCR results were consistent with western blotting results (Figure 5B). However, we found that the increases in the phosphorylated levels of STAT3 in the presence of LPS were significantly attenuated when HMGA1was knockdown, but silence of HMGA1 did not change the elevated level of COX-2 in cardiomyocytes with LPS stimuli (Figure 5C-D).

### COX-2 deletion abolished the effect of HMGA1 overexpression and STAT3 agonist reversed the role of HMGA1 silence

**Figure 6:**
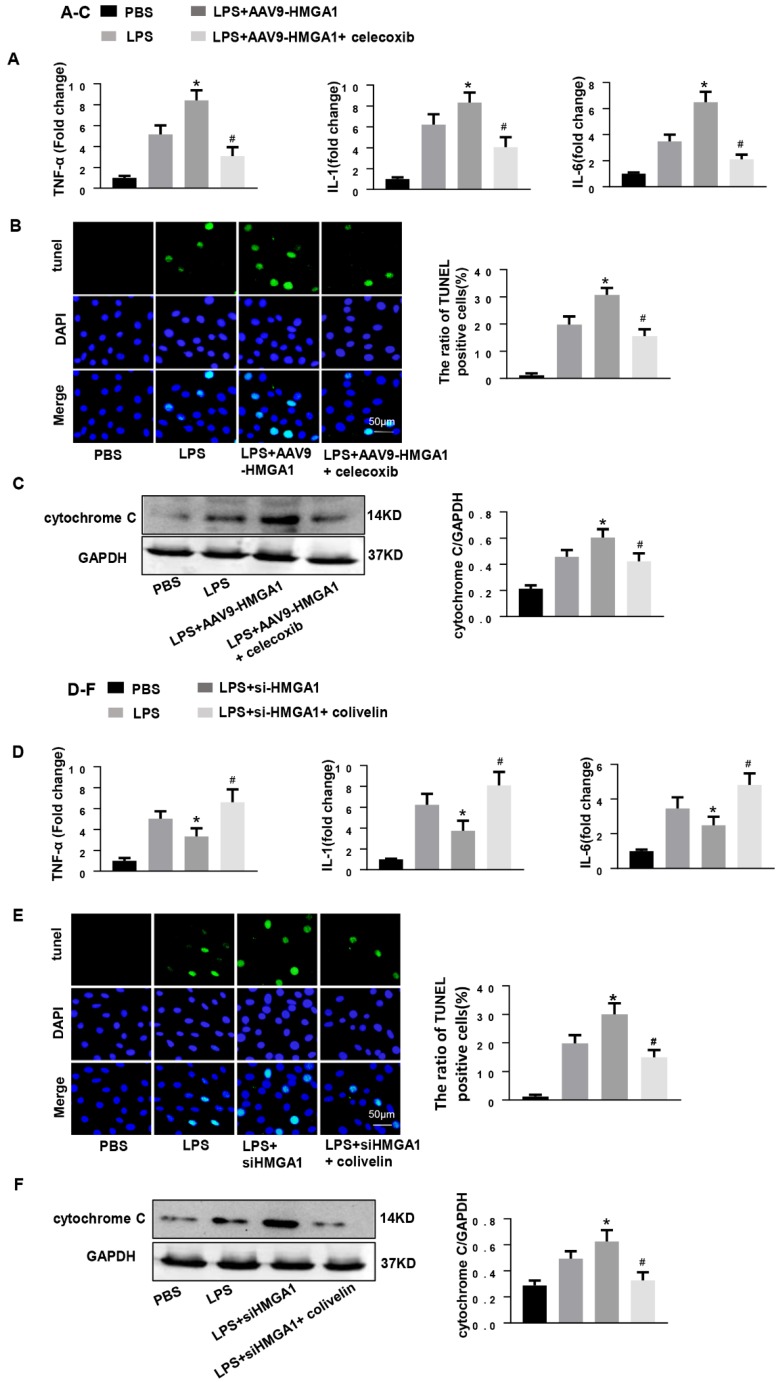
** COX-2 deletion abolished the effect of HMGA1 overexpression and STAT3 agonist reversed the role of HMGA1 silence. (A-C):** H9c2 cardiomyocytes were transfected with AAV9-HMGA1 then stimulated with LPS and treated with celecoxib (COX-2 inhibitor). (A)RT-PCR analyses for transcriptional level of inflammatory cytokines (TNF-α, IL-1, IL-6) in each group (n = 6). (B)TUNEL staining results for the detection of the apoptosis-positive cells in each group (n =6). (C) The protein expression level of pro-apoptotic cytochrome C was examined by western blotting and was normalized to GAPDH (n=6). *P<0.05 *vs.* the LPS group; *^#^P*<0.05 *vs.* the AAV9-HMGA1+LPS group. The data were shown as the mean±SD and were compared by one-way ANOVA with Tukey post hoc analysis. **(D-F):** H9c2 cardiomyocytes were processed with siHMGA1 then stimulated with LPS and treated with colivelin (a STAT3 agonist, 1uM). (D) RT-PCR analyses for transcriptional level of inflammatory cytokines (TNF-α, IL-1, IL-6) in each group (n = 6). (E)The apoptotic cells in each group were detected by TUNEL staining (n =6). (F) Western blotting result for the detection of cytochrome C and the expression level was normalized to GAPDH in each group (n=6). *P<0.05 vs. the LPS group; #*P*<0.05 vs. the si-HMGA1+LPS group. The data were shown as the mean±SD and were compared by one-way ANOVA with Tukey post hoc analysis.

Subsequently, we determined whether HMGA1 lost its pro-inflammatory and pro-apoptotic effect when COX-2 was blocked. We treated HMGA1 overexpressing H9c2 cardiomyocytes with celecoxib (a more selective COX-2 inhibitor, 10uM). As expected, the selective COX-2 inhibitor improved LPS-induced cardiac inflammation and apoptosis in HMGA1 overexpressing cells, which reflected by reducing the mRNA levels of TNF-α, IL-1, IL-6 and decreasing the number of apoptosis-positive cells (Figure 6A and 6B). Furthermore, the Cytochrome C, as detected by western blotting, was distinctly decreased in H9c2 cardiomyocytes with treatment of celecoxib compared to the control group after LPS stimuli (Figure 6C).

Then, H9c2 cardiomyocytes in the absence of HMGA1 were incubated with colivelin (a STAT3 agonist, 1uM). Importantly, the STAT3 agonist colivelin exerted pro-inflammatory and anti-apoptotic effects in HMGA1 silenced cells, which indicated by increasing the pro-inflammatory factor expression and decreasing the apoptosis level (Figure 6D and E). Besides, the expression of cytochrome C was also inhibited by colivelin with exposure of LPS (Figure 6F).

## Discussion

There is increasing evidence that HMGA1 protein plays a vital part in the development and progression of a plenty of cardiovascular diseases 16-18,21. The purpose of our study was to probe the influence of HMGA1 in SIC and its potential mechanism. We found that the expression of HMGA1 prominently increased in inflammatory cardiomyocytes based upon the experiments in vivo and *in vitro*. And overexpression of HMGA1 in mice and H9c2 cardiomyocytes after LPS stimuli resulted in the upregulation in inflammatory mediators, the increase in the number of cell apoptosis, and the deterioration in cardiac dysfunction. Interestingly, the effect of HMGA1 deficiency on LPS-stimulated H9c2 cardiomyocytes was partly consistent with the overexpression of HMGA1. Inhibition of HMGA1 expression reduced inflammation, but increased cell apoptosis. Overexpression of HMGA1 induced upregulation of COX-2, the increase in inflammatory cytokines as well as elevation of apoptosis, which was inhibited by celecoxib (a more selective COX-2 inhibitor). Additionally, the downregulation of STAT3 and the improvement of inflammation caused by HMGA1 deficiency were reversed by colivelin (a STAT3 agonist).

Here, to the best of our knowledge, it is evidenced that cytokines, especially IL‐1β, IL‐6, and TNF‐α, are major contributors in the initiation of SIC 22. It has been demonstrated that when endotoxin like LPS binds the receptor TLR4 that is expressed on cardiomyocytes and macrophages, it initiates the activation of NF‐κB and permits NF-κB translocating into nucleus 23. After undergoing LPS insult, both the macrophages and cardiomyocytes can release a great quantity of inflammatory mediator, such as MCP-1, GM-CSF, IL-1α, IL-1β, IL-6, IL-7, IL-8, and IL-12 22,23.These inflammatory cytokines could give rise to imbalance of calcium homeostasis 24, disturbance of energy metabolism 25, impairment of adrenergic signaling 26, and excess production of nitric oxide 27, all of which facilitate decreased contractility, diastolic dysfunction, impaired ejection fraction and reduced cardiac index. The high mobility group A1 (HMGA1) has a significant impact on the proliferation, differentiation, activation and recruitment of inflammatory cells, as well as the secretion of inflammatory mediators and cytokines 28. Prior researches have manifested that HMGA1 induces the expression of multiple genes participating in the inflammatory response, such as inducible nitric oxide synthase, cyclooxygenase-2, granulocyte-macrophage colony stimulation factor, IL-4 and IL-2 29,30. We found that Adeno-associated virus-mediated HMGA1 delivery elevated transcription level of inflammatory cytokines TNF-a, IL-1, IL-6 and inhibition of HMGA1 resulted in downregulated levels of inflammatory cytokines. The above data indicate that HMGA1 is a detrimental factor in myocardial inflammation.

LPS may activate the release of TNF-α or NO in an autocrine manner and increase the expression of AT1 receptors through the cardiac renin-angiotensin system to mediate apoptosis 31. Previous works have evidenced that two major signaling pathways mediate cardiomyocyte apoptosis in SIC, that is, mitochondria-dependent pathway that induces release of cytochrome C and then activates caspase-9 and the death-receptor-dependent pathway that brings about the activation of caspase-8 32, both of which ultimately lead to caspase-3 activation, promoting programmed cell death. In the Fedele study, the overexpression of HMGA1 can lead to apoptosis through caspase-3 activation in normal thyroid cells 33.Our finding surprisingly indicated that HMGA1-overexpressing and HMGA1-silence cells both undergone apoptosis through mitochondria pathway.

COX-2 accelerates the synthesis of prostaglandin E2 and promotes LPS-induced IL-1β secretion by increasing NF-κB activation, accompanying with enhancement of activating caspase-1 by damaging mitochondria 34. As a transcription factor, STAT3 has engaged in the regulation of numerous genes involved in cell apoptosis. Upon phosphorylation of tyrosine-705, STAT3 translocates into the nucleus and binds to specific DNA fragments to induce transcriptional up-regulation of anti-apoptotic proteins involving Bcl-xL and Hsp70 accompanied by down-regulation of pro-apoptotic genes including Bax through repressing the expression of the p53 tumor suppressor gene 35-38. In tumor studies, it was found that HMGA1 can up-regulate cyclooxygenase 2 (COX-2) expression by binding directly to its promoter 39. Furthermore, HMGA1 also binds directly to a conserved region of the STAT3 promoter in vivo by chromatin immunoprecipitation and activates transcription of the STAT3 in transfection experiments 40,41. Therefore, we detected the expression levels of COX-2 and STAT3 in cardiomyocytes when promotion or inhibition of HMGA1 expression under LPS stimulation. In our study, we found that overexpression of HMGA1 challenged with LPS affected COX-2 expression, in line with previous studies that HMGA1 activates COX-2 expression 39,42. In contrast, inhibition of HMGA1 expression altered STAT3 expression. This inconsistent may attribute to the different transcriptional target of HMGA1 under different expression level. Although it has been reported that COX-2 and STAT3 can regulate each other in other disease models, our results suggested that the expression of COX-2 in LPS-induved disease model may not depend on changes in STAT3. Our study found that when HMGA1 was overexpressed, it mainly affected COX-2 protein expression, and when HMGA1 expression was suppressed, it primarily regulated STAT3 expression. In addition, the expression of STAT3 and COX-2 was not positively related, so we thought COX-2 and STAT3 may not have a mutual regulating effect in this study, but that HMGA1 independently affected COX-2 and STAT3. Therefore, we hypothesized that HMGA1 played a complex role in the regulation of myocardial inflammation, and that this effect had a preference of affinity, while activating the pro-inflammatory factor COX-2 and the inflammatory signal STAT3. Further study is still needed to elucidate this selection bias of HMGA1.

A variety of extracellular and intracellular signals can induce different post-translational modifications (PTMs) of HMGA1 protein, which can significantly change the affinity of HMGA1 for DNA and chromatin substrates, thereby affecting the nuclear function of HMGA1 43,44. It is reported that phosphorylation of HMGA1 reduces its ability to bind to DNA and activates transcription 45. In future studies, we will further explore phosphorylation of HMGA1 and its role in SIC.

In short, our research indicated that the activation of HMGA1 promoted cardiac inflammation and apoptosis induced by LPS via activation of COX-2, whereas silence of HMGA1 inhibited cell inflammation but aggravated apoptosis of cells through phosphorylation of STAT3.

## Figures and Tables

**Table 1:** Primer sequences used in this study

Species	Target genes		Sequences (5' - 3')
Rat	HMGA1	Forward	CGCTGGTAGGGAGTCAGAAG
		Reverse	CTCCAGTTTCTTGGGTCTGC
Rat	TNF-α	Forward	AGCATGATCCGAGATGTGGAA
		Reverse	TAGACAGAAGAGCGTGGTGGC
Rat	IL-1	Forward	GGGATGATGACGACCTGCTAG
		Reverse	ACCACTTGTTGGCTTATGTTCTG
Rat	IL-6	Forward	GTTGCCTTCTTGGGACTGATG
		Reverse	ATACTGGTCTGTTGTGGGTGGT
Rat	STAT3	Forward	CACCCATAGTGAGCCCTTGGA
		Reverse	TGAGTGCAGTGACCAGGACAGA
Rat	COX-2	Forward	ACACTCTATCACTGGCATCC
		Reverse	GAAGGGACACCCTTTCACAT
Rat	GAPHD	Forward	GACATGCCGCCTGGAGAAAC
		Reverse	AGCCCAGGATGCCCTTTAGT
Mice	HMGA1	Forward	AGCAGGAAAAGGATGGGACT
		Reverse	CTCCAGTTTCTTGGGTCTGC
Mice	TNF-α	Forward	ACGGCATGGATCTCAAAGAC
		Reverse	GTGGGTGAGGAGCACGTAGT
Mice	IL-1	Forward	CTCGCAGCAGCACATCAACAAG
		Reverse	CCACGGGAAAGACACAGGTAG
Mice	IL-6	Forward	GAGGATACCACTCCCAACAGACC
		Reverse	AAGTGCATCATCGTTGTTCATACA
Mice	GAPHD	Forward	GACATGCCGCCTGGAGAAAC
		Reverse	AGCCCAGGATGCCCTTTAG
